# Residual disease and immune infiltration as a new surrogate endpoint for TNBC post neoadjuvant chemotherapy

**DOI:** 10.18632/oncotarget.27081

**Published:** 2019-07-23

**Authors:** Stephen L. Luen, Roberto Salgado, Sherene Loi

**Affiliations:** Peter MacCallum Cancer Centre, Melbourne, Australia

**Keywords:** triple negative breast cancer, tumor infiltrating lymphocytes, neoadjuvant chemotherapy, prognosis

Triple negative breast cancer (TNBC) is distinguished from other breast cancer subtypes by its lack of therapeutic targets, aggressive biology, and poorest survival rates. In the early breast cancer setting, cytotoxic chemotherapy remains the mainstay of systemic treatment options. Multiple clinical trials and meta-analyses have demonstrated that patients who achieve a pathological complete response (pCR) to neoadjuvant chemotherapy (NAC) have an excellent prognosis [1]. Therefore, NAC is now extensively used for early stage TNBC (and HER2-positive breast cancer) to improve prognostic stratification, but also in the setting of residual disease, to enrich for high risk, treatment resistant patients that may benefit from treatment escalation or change of systemic therapy [2]. Reported rates of pathologic complete response (pCR) to neoadjuvant chemotherapy (NAC) regimens in TNBC are typically in the vicinity of 40–60% depending on patient selection and chosen treatment regimen. Higher levels of pre-treatment tumor-infiltrating lymphocytes (TIL) are associated with high rates of pCR suggesting that the presence of robust host immunity contributes to chemotherapy response [3, 4]. This suggests that substantial biological heterogeneity exists within the TNBC subtype.

**Figure 1:**
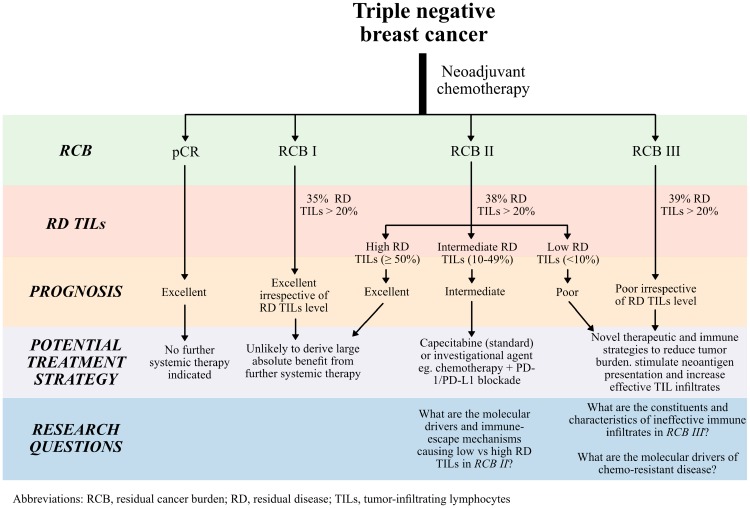
Flow diagram demonstrating the potential clinical utility of residual cancer burden (RCB) and residual disease tumor-infiltrating lymphocytes in patients with residual disease after neoadjuvant chemotherapy for triple negative breast cancer. Future research questions are also proposed.

We rationalised that the most clinically relevant heterogeneity exists in patients with tumors that do not achieve a pCR, and that biomarkers that refine prognostic estimates and improve biological understanding in this population might help guide the development of hypothesis-driven clinical trials in the future. We recently reported results from our analysis that combined four data series of patients with TNBC who received NAC and did not achieve pCR, with a key focus on two highly prognostic biomarkers – residual disease TILs, and residual cancer burden (RCB) [5]. This editorial highlights some of the findings from this report, discusses potential clinical implications, and future areas of research (Figure 1).

Our study demonstrated that both RCB and residual disease TILs are powerful prognostic markers that can further refine prognostic estimates in this population. The best survival outcomes were observed for patients with minimal residual disease burden (RCB class I). Other studies have demonstrated that patients achieving RCB class I after NAC have a similar prognosis to those who achieve pCR [6, 7]. Concordant with this, the 5 year overall survival (OS) estimate for patients achieving RCB I in our cohort was high at 91% (95% confidence interval [CI] 81–100%), and hence no significant prognostic influence of residual disease TILs was observed. Given the excellent long term prognosis we hypothesize that these patients are unlikely to gain large absolute benefit from the addition of further adjuvant systemic therapy [2].

On the other hand, the worst disease outcomes were observed for patients with extensive residual disease burden (RCB class III), with a 5 years OS estimate of 31% (95% CI 24–40). The majority of these patients (82%) experienced disease recurrence, of which 72% had a recurrence which occurred early (within 12 months of definitive surgery). In our cohort, the median OS (from the time of recurrence) for patients who developed an early recurrence in our cohort (irrespective of RCB class) was a dismal 6.1 months (95% CI 5.2–8.8). This highlights an urgent need for further research into these poor prognosis patients who are essentially chemo-refractory. Notably, the efficacy of PD(L)-1 blockade in this population remains unclear as patients with an early recurrence were not eligible for the recently published IMpassion130 study [8]. Interestingly, our study found no significant positive prognostic influence of higher residual disease TILs for patients with RCB class III, suggesting either the immune infiltrates are significantly suppressed, ineffective, or exhausted, despite these being predominantly CD8^+^ T cells [9, 10]. Further research will be required to understand the biological mechanisms underlying chemo-refractory primary TNBC, as well the exact T cell phenotype, however it is extremely likely that novel therapeutic strategies, including immunotherapy combinations, will be needed to both effectively reduce tumor burden, and stimulate active anti-tumor immune responses. We hypothesize that single agent PD(L)-1 inhibition will be ineffective in this group.

The positive prognostic influence of residual disease TILs was greatest in patients with moderate residual disease burden (RCB class II), whereby prognosis could be stratified to outcomes similar to that of RCB class I and RCB class III depending on the quantity of residual disease TILs. The significant prognostic influence of residual disease TILs, and the heterogeneity in disease outcomes in this setting suggests that adjuvant immunotherapies, such as with PD(L)-1 blockade with chemotherapy could potentially result in survival gains for these patients. Further research into the tumor intrinsic mechanisms underpinning the presence or absence of residual disease TILs in RCB class II, as well as the immune checkpoint molecules that are frequently expressed on T cells, will further help refine therapeutic strategies for these patients.

Beyond improved prognostic stratification, we believe that there is a need to find a more refined biomarker surrogate for patient survival than pCR alone. Therapeutic strategies that have led to significantly increased pCR rates to neoadjuvant treatments have not uniformly lead to significant survival benefits for patients [1]. We rationalise that a composite clinical trial endpoint for neoadjuvant trials that encompasses both residual disease (RCB), as well as immune response to therapy may function as a better predictor of survival benefit. To develop such a surrogate marker we would require large randomized clinical trials collecting pCR status, RCB class, and TIL evaluation, alongside event-free and OS data, to understand how changes in these parameters correlate to survival. A more accurate short-term surrogate endpoint would be the Holy Grail for rapid and efficient drug development. The prognostic significance of residual disease TILs, as well as the value of early biopsy evaluating on-treatment TILs are also worthy of exploration in other cancer types, however the value of these biomarkers in predicting outcome may differ by cancer type and utilized therapy.

In summary, we believe our data can be used to improve prognostic stratification, help refine target populations for research, and contribute to the design of future neoadjuvant clinical trials.
